# Neuroprotective effects of *Psoralea corylifolia* Linn seed extracts on mitochondrial dysfunction induced by 3-nitropropionic acid

**DOI:** 10.1186/1472-6882-14-370

**Published:** 2014-10-03

**Authors:** A-Rang Im, Sung-Wook Chae, Gui jun Zhang, Mi-Young Lee

**Affiliations:** KM-Based Herbal Drug Development Group, Korea Institute of Oriental Medicine, 1672 Yuseongdae-ro, Yuseong-gu, Daejeon, 305-811 Korea; School of Chinese Materia Medica, Beijing University of Chinese Medicine, Beijing, 100102 China

**Keywords:** *Psoralea corylifolia* Linn seed, 3-nitropropionic acid, Mitochondrial dysfunction, Cellular bioenergetics

## Abstract

**Background:**

Mitochondrial dysfunction has been implicated in neuronal apoptosis associated with neurodegenerative diseases such as Huntington’s disease (HD). Animals that are administered 3-nitropropionic acid (3-NP), a mitochondrial toxin that specifically inhibits complex II of the mitochondrial electron transport chain, manifest HD-like symptoms.

**Methods:**

*Psoralea corylifolia* Linn seed extracts against 3-NP induced mitochondrial dysfunction in cultured rat pheochromocytoma (PC12) cells, which are used for neurobiological studies.

**Results:**

In this study showed that 3-NP-treated PC12 cells had decreased ATP levels, lower cellular oxygen consumption, and reduced mitochondrial membrane potential than those of untreated PC12 cells. *Psoralea corylifolia* Linn seed extracts stimulated mitochondrial respiration with uncoupling and induced an increased bioenergetic reserve capacity. Furthermore, PC12 cells pretreated with *P. corylifolia* Linn seed extracts significantly attenuated 3-NP-induced cell death, reduced ATP levels, and lowered the mitochondrial membrane potential.

**Conclusions:**

These results demonstrate that *P. corylifolia* Linn seed extracts have a significant protective effect against 3-NP induced cytotoxicity. Thus, our results indicate that *P. corylifolia* Linn seed extracts may have potential applications as therapeutic agents for treating neurodegenerative disease.

## Background

*Psoralea corylifolia* Linn is an herbaceous legume found in India, China, and Korea, and it is commonly used in traditional Chinese medicine [1]. *P. corylifolia* extracts have been shown to possess several therapeutic properties, including anti-tumor, anti-inflammatory, and radiomodulatory properties, and have been shown to alleviate osteoporosis in rats [2–5]. A previous study showed that Δ^3^-2-hydroxybakuchiol isolated from *P. corylifolia* has dopaminergic neuroprotective effects *in vitro* and has antiparkinsonian-like effects *in vivo* [6]. Previous mouse model studies have shown that *P. corylifolia* seed extract components, psoralidin and furocoumarins, possess potent anti-depressant properties [7–9].

3-nitropropionic acid (3-NP) is a specific inhibitor of mitochondrial respiratory complex II and can cause HD -like symptoms in animals upon ingestion [10]. A previous study showed that complex II inhibition by 3-NP resulted in mitochondrial fragmentation and neuronal cell death via *N*-methyl-d-aspartate and reactive oxygen species (ROS)-dependent pathways [11].

Brain neuron cells have higher sensitivity to oxidative stress than other cells owing to their dependence on oxidative phosphorylation as the primary energy source [12]. Mitochondria play an important role in apoptosis and neuronal degeneration. Due to the constant exposure to high levels of energy and oxygen, mitochondria are vulnerable to oxidative stress damage. Mitochondrial electron leakage causes oxidative stress that can lead to cell death. The mechanisms underlying oxidative damage- induced neuronal death are poorly understood, and whether mitochondrial defects are the primary cause of toxicity or a secondary response to the damage remains unknown [13]. However, impaired respiratory chain activity or failure of mitochondrial function has been implicated in the pathogenesis of several mitochondrial disorders including neurodegenerative disorders [14].

Cell respiratory capacity declines with age, and this decline is exaggerated in neurodegenerative mitochondrial diseases. Mitochondria integrate extracellular signals, carry out essential cellular functions, and determine neuronal survival and death. Since, mitochondrial metabolism is recognized as the primary source of ROS, mitochondrial biogenesis is expected to increase oxidative stress in neurons [15]. A previous study showed that a respiratory reserve could provide a valuable therapeutic target for treating mitochondrial disorders and developing pharmacologic approaches that tap into this respiratory reserve could prove useful for treating neurodegenerative disorders [16].

In this study, we investigated the neuroprotective effect of *P. corylifolia* seed extracts against 3-NP induced mitochondrial dysfunction in cultured rat pheochromocytoma (PC12) cells, which are widely used for neurobiological studies. We particularly studied the impact of *P. corylifolia* seed extracts on mitochondrial toxins and on mitochondrial bioenergetic function.

## Methods

### Materials

3-NP, oligomycin, and rotenone were purchased from Sigma (St. Louis, MO, USA). RPMI 1640 and fetal bovine serum (FBS) were purchased from Gibco BRL (Grand Island, NY). CellTiter Aqueous One Solution Cell proliferation assay (MTS) kits were purchased from Promega Co. (Madison, USA). A luminescence ATP detection kit (PerkinElmer, Waltham, MA, USA) and JC-1 mitochondrial membrane potential detection kit (Biotium, Hayward, CA) were used. Mitotracker®, Image-iT live green ROS detection kit and MitoSOX™, Annexin-V, and propidium iodide (PI) double staining kit were purchased from Invitrogen Molecular Probes (San Diego, CA). XF-24 cell culture microplates, extracellular flux assay kits, XF calibrant, and XF assay medium were purchased from Seahorse Bioscience (Billerica, MA).

### Preparation of *P. corylifolia*seed extracts

*P. corylifolia* seed was purchased from Kwangmyungdang Medicinal herbs (Ulsan, Korea). A voucher specimen (KIOM111930, KIOM211930) has been deposited at the herbarium of the Department of Aging Research Lab., Korea Institute of Oriental Medicine, South Korea. Aqueous extracts of *P. corylifolia* seed were prepared by 300 g of powdered plant material was mixed with 3 L of distilled water in a flask and sonicating for 2 h. The process was repeated three times. The suspension was lyophilized of water extract. A 80% ethanol extract was prepared by sonicating the dried ground powder suspended in 80% ethanol solvent (v/v% in water) and the suspension was processed as described for the aqueous extract.

### Cell viability

PC12 cells (Korean Cell Line Bank, Korea) were cultured in RPMI 1640 with 10% fetal bovine serum (FBS) at 37°C with 5% CO_2_. PC12 cells were plated at a density of 1 × 10^4^ cells/well with 200 μl RPMI 1640 containing 10% FBS in a 96-well collagen IV-coated plates and were incubated at 37°C for 24 h. *P. corylifolia* seed extracts prepared with water (PCWE) and with 80% ethanol (PCEE) added to the cell culture plates followed by incubation at 37°C for 24 h. Cell viability was determined by performing an MTS test that assesses bioreduction of MTS to formazan. The plates were assayed at 490 nm by using a microplate fluorometer (Molecular Devices, Sunnyvale, CA, USA). For determining the protective effects of the extract in rotenone-induced PC12 cells, samples of various concentrations were treated in a 96-well collagen IV-coated plate at 24 h after adding 25 μM of 3-NP. After 3 h, the medium was removed and the MTS assay was performed to assess the cell viability.

### ATP measurement

Various concentrations of extracts were added to cells in a 96-well white plate for 1 h before adding 25 μM of 3-NP and incubating for 3 h. Total cellular ATP content was determined by using a luminescence ATP detection kit and a in accordance with the manufacturer’s instructions (PerkinElmer, Waltham, MA, USA). The total cellular ATP content was determined by running an internal standard and expressed as the percentage of untreated cells (control).

### XF-24 metabolic flux analysis

Oxygen consumption rate (OCR) was measured with a Seahorse XF24 Extracellular Flux Analyzer (Seahorse Bioscience, Billerica, MA). PC12 cells were seeded in Seahorse XF-24 plates at a density of 5 × 10^4^ cells/well. Cells were treated with the sample in a 96-well white plate 24 h before adding 25 μM of 3-NP. On the day of the metabolic flux analysis, cells were switched to unbuffered DMEM (DMEM base medium supplemented with 25 mM glucose, 2 mM sodium pyruvate, 31 mM NaCl, 2 mM GlutaMax, pH 7.4) and incubated at 37°C in an incubator with no CO_2_ for 1 h. Three readings were taken after each addition of mitochondrial inhibitor before injection of the subsequent inhibitors. The mitochondrial inhibitors used were ATP synthase inhibitor oligomycin (final concentration: 1 μg/ml), the proton ionophore carbonylcyanide p-trifluoromethoxyphenylhydrazone (FCCP; final concentration 1 μM), and complex I inhibitor rotenone (final concentration, 1 μM). Mitochondrial function parameters were determined using mitochondrial inhibitors as modulators to determine the number of bionergetic and mitochondrial function parameters, including basal respiration, ATP turnover rate, proton leak, and maximal and spare respiratory capacity. OCR was automatically calculated and recorded using the Seahorse XF-24 software.

### Mitochondrial membrane potential

Cells were treated with samples in a 96-well white plate for 24 h before adding 25 μM of 3-NP. MMP of PC12 cells was measured by using a JC-1 mitochondrial membrane potential assay kit (Biotium, Hayward, CA, USA) according to the manufacturer’s instructions. The plates were then incubated at 37°C for 20 min after the addition of 100 μl of 1 × JC-1 reagent into the wells. Red fluorescence (excitation, 550 nm; and emission, 600 nm) and green fluorescence (excitation, 485 nm and emission, 535 nm) were determined using a Softmax Pro 5 fluorescence plate reader (Molecular Devices, Sunnyvale, CA, USA). The ratio of red to green fluorescence of dead cells and of cells undergoing apoptosis is lower than that of the healthy cells. For confocal microscope analysis, 1× JC-1 was added to treated cells and cells were incubated for 15 min at 37°C. Cells were imaged using a confocal microscope (FV10i-LIV, Olympus, Tokyo, Japan). In live non-apoptotic cells, mitochondria appeared red following aggregation of the JC-1 reagent. The excitation of the red aggregates occurred at 559 nm and emission was at 570–620 nm. In both the apoptotic cells and dead cells, the dye remained in its monomeric form and appeared green with excitation at 473 nm and emission at 490–540 nm.

### Mitochondrial superoxide

MitoSOX™ Red reagent was used for determining the superoxide levels. MitoSOX™ does not react with ROS and reactive nitrogen species within the cell, but it selectively targets the mitochondria after permeating the cells and rapidly reacting with the superoxides within the mitochondrial matrix. Varying concentrations of samples were added to the cells plated in the 96-well white plate 1 h before adding 25 μM of 3-NP for 3 h. Cells were incubated with 5 μM MitoSOX™ Red for 20 min at 37°C. MitoSOX™ Red has excitation/emission maxima of approximately 510/580 nm and the fluorescence was measured by using the fluorescence plate reader Softmax Pro 5 (Molecular Devices). A confocal microscope (Olympus) was used for visual analysis of the cells.

### Confocal microscopy analysis

PC12 cells were plated at a density of 5 × 10^4^ cells on a chamber slide. Cells were treated with samples at concentrations that ranged between 10–100 μg/ml for 24 h before adding 25 μM of 3-NP. Cells were incubated for 45 min with 10 μM Mitotracker® Red and 1 μg/ml 4′,6-diamidino-2-phenylindole (DAPI) for detecting the mitochondria. Cells were visualized under a confocal microscope (Olympus) and the fluorescence emission and excitation was at 598 nm and 578 nm, respectively. DAPI fluorescence was determined at an excitation wavelength of 359 nm, and emission was detected at 461 nm.

### Statistics

All data are expressed as mean ± standard deviation of at least three independent experiments and analyzed by one-way ANOVA and by multiple comparisons that were performed by using the Student’s *t*-test. A p-value of less than or equal to 0.05 was considered significant.

## Results

### Protective effect of PCWE and PCEE on 3-NP treated PC12 cells

**Figure 1:**
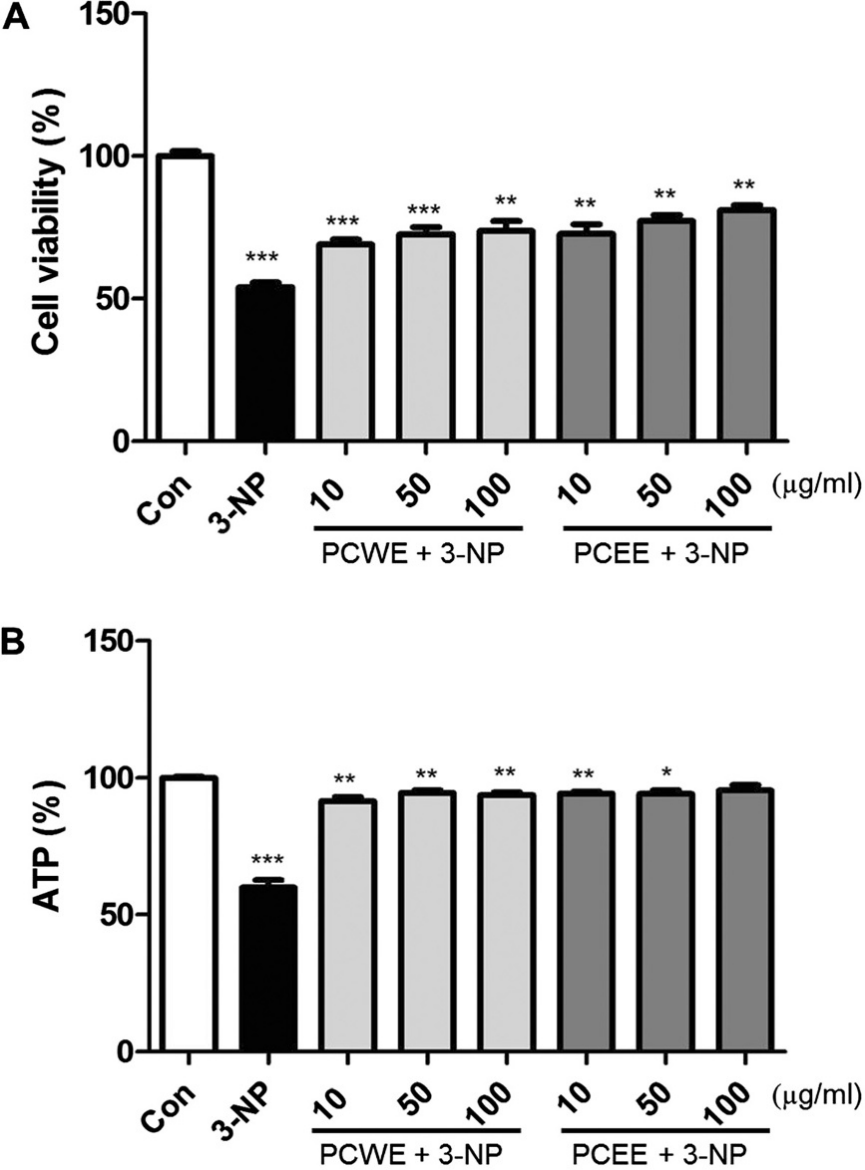
**Protective effect of** ***P. corylifolia*** **seed extracts. (A)** Protective effect of *P. corylifolia* seed water extract (PCWE) and *P. corylifolia* seed ethanol extract (PCEE) pretreatment in 3-nitropropionic acid (3-NP) treated pheochromocytoma (PC12) cells. Data are reported as a percentage of control cell viability. **(B)** Effects of PCWE and PCEE on total cellular ATP levels. ATP levels were measured by using ATPlite^TM^ luminescence-based assay. Data are reported as a relative percentage of control. * *p* < 0.05, ** *p* <0.01, and *** *p* <0.001.

In this study, extraction yield of PCWE was 20.6% and PCEE was 20.4%. We first examined the cytoprotective effect of the sample against 3-NP induced cell death by using MTS assay. The dose of 3-NP and the exposure period to 3-NP that reduce the cell viability to 50% were determined by using various 3-NP concentrations that ranged between 10 μM to 1 mM (data not shown). The data indicated that PC 12 cells treated with 25 μM 3-NP for 3 h had cell viability of 54.1% as compared to the cell viability of 100% of the control cells. Pretreatment with PCWE for 24 h followed by 3-NP exposure resulted in higher viability (viability of triplicates: 69.0%, 72.6%, and 73.7%) than that of 3-NP exposed PC12 cells that were not pretreated with PCWE. Furthermore, pretreatment with PCEE for 24 h followed by 3-NP exposure also resulted in higher cell viability (viability of triplicates: 72.6%, 77.2%, and 81.1%) than that of 3-NP exposed PC12 cells that were not pretreated with PCEE (Figure 1A).

### Effects of PCWE and PCEE on total cellular ATP levels

To determine whether the samples had an impact on energy production, we measured ATP concentrations by using the ATP lite assay kit. The cellular ATP levels of 3-NP treated cells after 3 h incubation were significantly decreased (59.8%) from those of the control cells (100%). As shown in Figure 1B, the ATP levels of 3-NP treated group were lower than those of the sample-pretreated group.

### Bioenergetics analysis and mitochondrial function test

**Figure 2:**
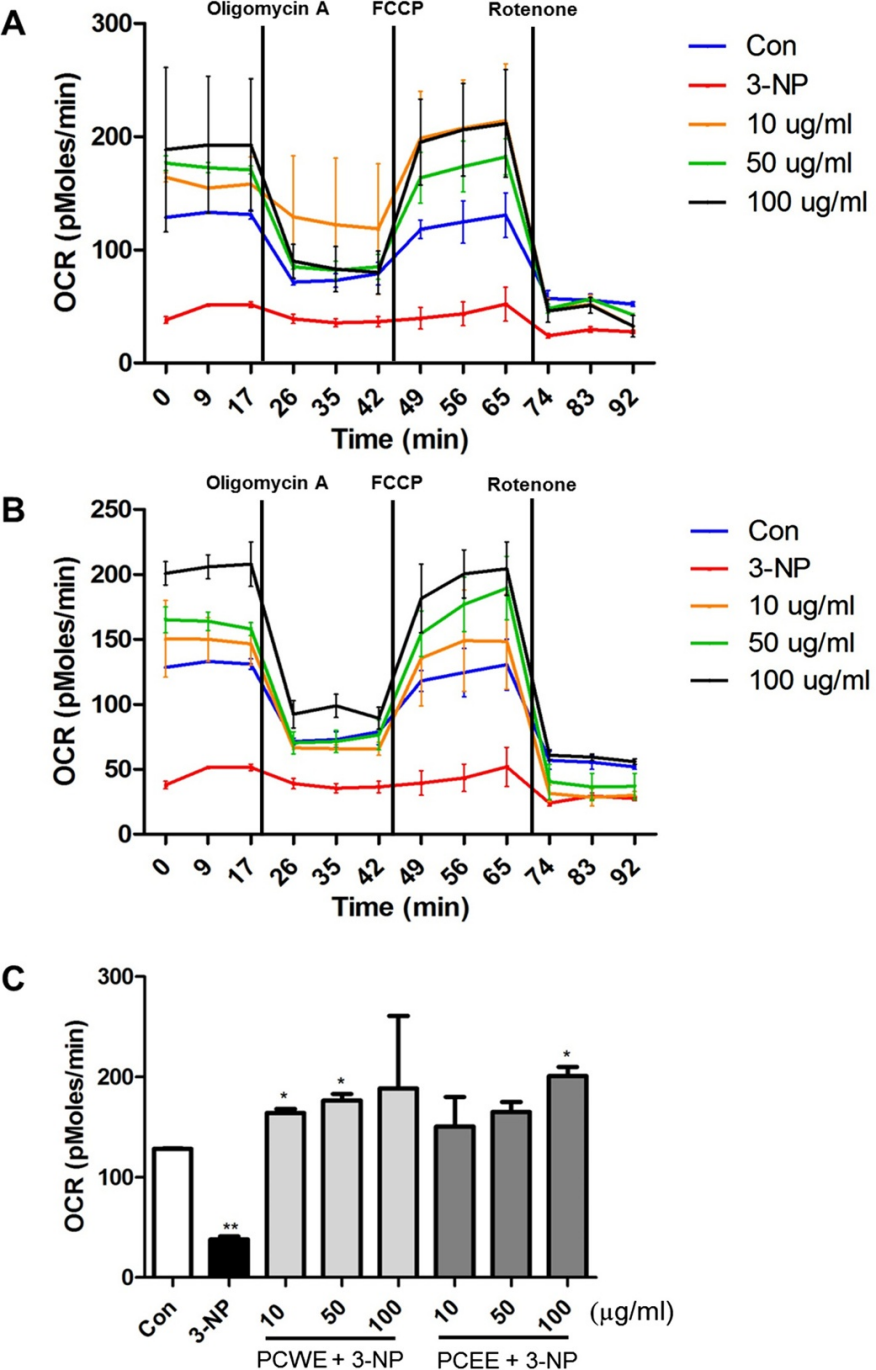
**Mitochondrial respiration of PC12 cells Oxygen consumption rate (OCR) was monitored in control cells, 3-NP treated cells, and in cells that were pretreated with PCWE or PCEE followed by 3-NP treatment. (A)** Cells were pretreated with PCWE at concentrations of 10, 50, and 100 μg/ml for 24 h followed by 25 μM 3-NP treatment for 3 h. **(B)** Cells were pretreated with PCEE at concentrations of 10, 50, and 100 μg/ml for 24 h followed by treatment with 25 μM 3-NP for 3 h. **(C)** Basal respiration was calculated from OCR. Vertical lines indicate time of addition of mitochondrial inhibitors A: oligomycin (1 μg/ml), B: FCCP (1 μM), or C: rotenone (1 μM). * *p* < 0.05, and ** *p* <0.01.

Seahorse cellular bioenergetic analyzer was used to examine the effects of PCWE and PCEE on mitochondrial respiration and cellular glycolysis in PC12 cells. Oligomycin A (complex V inhibitor), FCCP (uncoupler), and rotenone (complex I inhibitor) were sequentially applied to determine the action of PCWE and PCEE on the electron transport chain (Figure 2). The data showed that the OCR was increased by the pretreatment of PCWE (Figure 2A) and PCEE (Figure 2B). We found that 3-NP treatment decreased the mitochondrial OCR to 38 pMol/min compare to control cells (129 pMol/min) of the basal level (Figure 2C) that indicate 3-NP exposure inhibited basal mitochondrial respiration. However, 3-NP treated cells that were pretreated with either 100 μg/ml PCWE or PCEE to prevent intracellular acidification had OCR of 189 pMol/min and 201 pMol/min, respectively. Thus, the extract pretreated cells had mitochondrial OCR that were comparable to that of the control cells that were not exposed to the extracts or 3-NP. In a previous study, the coupling efficiency and spare respiratory capacity under these conditions were estimated by using oligomycin and FCCP, respectively [17]. In this study, after measurement of the basal OCR, oligomycin was injected into all samples to inhibit the ATP synthase (complex V). PC12 cells that were pretreated with 3-NP showed a substantial decrease in OCR. However, the OCR of oligomycin-treated cells that were also pretreated with PCWE and PCEE can be ascribed to both proton leakages across the mitochondrial membrane and to the utilization of the mitochondrial membrane potential for ion or substrate transport. To determine the maximal OCR that the cells can sustain, the proton ionophore (uncoupler) FCCP was injected after oligomycin treatment. FCCP addition resulted in stimulation of OCR due to increased proton permeability of the mitochondrial inner membrane that resulted in an OCR that was unconstrained by the mitochondrial membrane potential. Thus, the FCCP-treated cell mitochondrial respiration reached maximal capacity and was higher than that of the control cells. Rotenone was injected lastly to inhibit electron flux through complex I, which causes OCR suppression.

### Mitochondrial membrane potential

**Figure 3:**
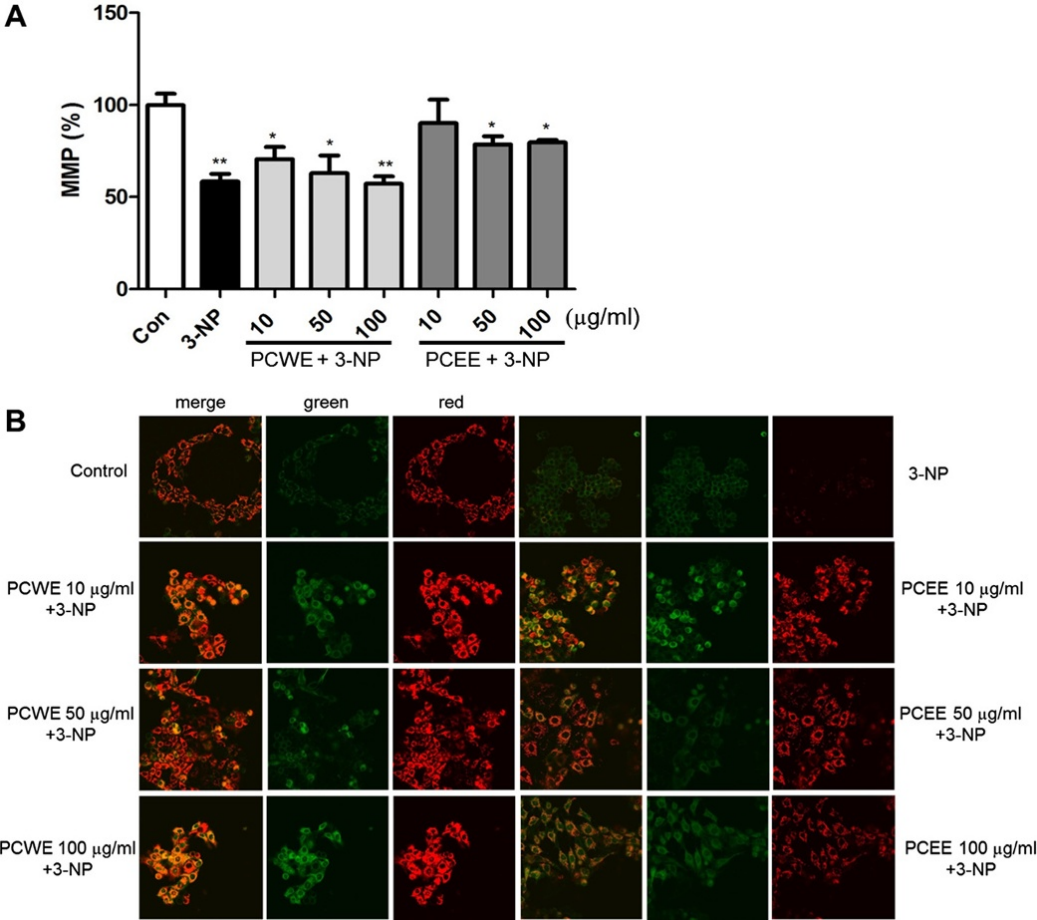
**Mitochondrial membrane potential (MMP) of the PC12 cells measured by JC-1 as an indicator of mitochondrial function. (A)** Red fluorescence intensity (excitation, 550 nm; emission, 600 nm) and green fluorescence intensity (excitation, 485 nm; emission, 535 nm) were determined by using a fluorescence plate reader. **(B)** Confocal images show JC-1 fluorescence (60 × 3.5). Data are reported as a percentage of the control. * *p* < 0.05, and ** *p* <0.01.

We next examined the effect of PCWE and PCEE treatment on 3-NP-induced cell mitochondrial membrane potential, which was measured by performing JC-1 staining. The red signal of 3-NP significantly decreased, and consequently, the red/green signal ratio also decreased as indicated by JC-1 staining (Figure 3). 3-NP increased the mitochondrial membrane potential in the initial stages of the experiment as indicated by the alterations in JC-1 dye aggregation. 3-NP treatment for 3 h caused a significant reduction in JC-1 ratio, as indicated by the depolarized MMP, and inhibited a significant increase in MMP with 10 μg/ml PCEE treatment, as indicated by 88.7% increase in red JC-1 fluorescence (Figure 3A). As shown in figure 3B, cells pretreated with 3-NP had lower red signal intensity than the control cells. The MMP of PC12 cells that were treated with PCWE or PCEE showed a gradual recovery, as indicated by the reappearance of red-stained mitochondria.

### Confocal microscopy analysis of mitochondrial superoxide levels and mitochondria

**Figure 4:**
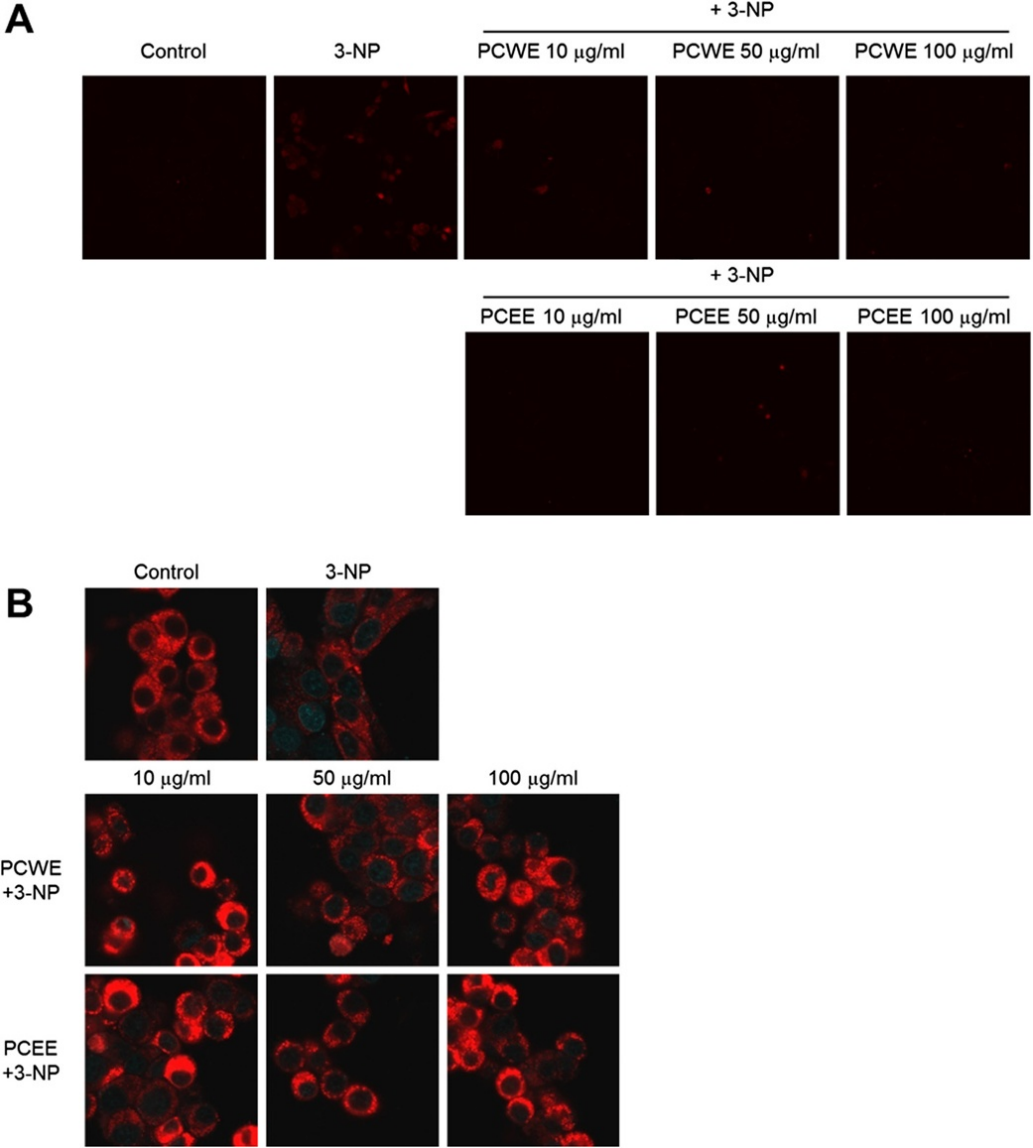
**Confocal microscopy analysis of mitochondrial superoxide production and mitochondria. (A)** MitoSOX™ Red staining followed by confocal microscopy analysis (60 × 3.5) was used to measure mitochondrial superoxide production. **(B)** Mitotracker® Red fluorescence stained mitochondria. Merged images of cells stained with Mitotracker® Red with 4',6-diamidino-2-phenylindole (DAPI) blue fluorescence stained cells were used to determine colocalization by using confocal microscopy (10 × 3.5). All data are reported as a percentage of control. * *p* < 0.05.

For detection of mitochondrial superoxide, we used MitoSOX™ Red mitochondrial superoxide reagent. Cellular MitoSOX™ fluorescence intensities were assessed by confocal microscopy (Figure 4A). 3-NP treated cells had higher mitochondrial superoxide production signals than that of the PCWE or PCEE pretreated cells. These results show that PCWE and PCEE protected the cells from 3-NP-induced mitochondrial superoxide production.MitoTracker ®, a red fluorescent dye, was used to stain PC12 cell mitochondria. Confocal microscope analysis revealed that the stained mitochondria in control cells were evenly distributed around the cell (Figure 4B). However, the 3-NP induced cells showed low red fluorescence intensity in mitochondria, indicating depolarization of the inner mitochondrial membrane.

## Discussion

*P. corylifolia* extracts have been shown to possess several therapeutic properties including neuroprotective effects. In previous researches, in vitro dopaminergic neuroprotective and in vivo antiparkinsonian-like effects of Delta 3,2-hydroxybakuchiol isolated from *Psoralea corylifolia* was investigated [18]. Also, seeds of *P. corylifolia* was found to strongly inhibit dopamine uptake by dopamine transporter heterogeneously expressed cells and noradrenaline uptake by noradrenaline transporter [19].

Neurodegenerative diseases are associated with mitochondrial metabolism impairment that leads to increased ROS and mitochondrial dysfunction. The mechanisms underlying HD development have been associated with mitochondrial dysfunction, which plays a critical role in this process. The HD brain tissues have decreased levels of electron transport chain complexes II and III [20, 21]. Therefore, mitochondrial respiratory complex II inhibition may play an important role in HD. Administration of 3-NP, a complex II inhibitor, induces symptoms that are associated with neurodegenerative diseases such as HD. Animals that were orally administered 3-NP developed HD-like neuropathology [22, 23]. 3-NP treatment induced cell death both by apoptosis and necrosis in PC 12 cells.

One of the modes of action of 3-NP-mediated toxicity involves energy depletion by inhibition of the complex II of the electron transport chain. Neuronal cells maintain a bioenergetic capacity that is sufficient to meet physiological energy demands, with a reserve or spare capacity that can be utilized by the cells under stress [17]. The neuroprotective benefits of *P. corylifolia* Linn seed could be partially attributed to 3-NP-induced enhancement of mitochondrial function. Extracellular flux analysis allows determination of oxygen consumption and extracellular acidification in cells. 3-NP inhibited OCR, indicating mitochondrial dysfunction. *P. corylifolia* Linn seed extracts treatment increased the mitochondrial respiratory capacity as indicated by the OCR value. The basal OCR was decreased in 3-NP treated cells and was increased in pretreated extracts and 3-NP-exposed cells.

Measurement of MMP and mitochondrial superoxide generation further demonstrated the impact of *P. corylifolia* Linn seed extracts. Our results indicated that *P. corylifolia* Linn seed extract treatment increased the MMP, correlated with the capacity for ATP production, and decreased the mitochondrial superoxide generation. Furthermore, a previous study showed that inhibition of the electron transport chain by 3-NP leads to the production of mitochondrial superoxide and the disruption of mitochondrial respiration caused by 3-NP results in the production of less ATP as well as more ROS [24].

## Conclusions

The results from this study suggest that *P. corylifolia* Linn seed extracts induced production of ATP and MMP and these decreased the mitochondrial superoxide levels. These findings suggest that *P. corylifolia* Linn seed could be potentially used as a therapeutic agent for treating neurodegenerative diseases. Future studies for identification of the active chemical moiety of the extracts may serve as a starting point for developing neuroprotective therapeutics to treat neurodegenerative disease.
